# *In silico* Analysis of Gamma-Secretase-Complex Mutations in Hidradenitis Suppurativa Demonstrates Disease-Specific Substrate Recognition and Cleavage Alterations

**DOI:** 10.3389/fmed.2019.00206

**Published:** 2019-09-19

**Authors:** John W. Frew, Kristina Navrazhina

**Affiliations:** ^1^Laboratory of Investigative Dermatology, The Rockefeller University, New York, NY, United States; ^2^Weill Cornell/Rockefeller/Sloan Kettering Tri-Institutional MD-PhD Program, Weill Cornell University, White Plains, NY, United States

**Keywords:** Hidradenitis Suppurativa, Alzheimer's disease, gamma secretase complex, nicastrin, pre-senilin

## Abstract

**Background:** Familial Hidradenitis Suppurativa and Familial Alzheimer's Disease are both associated with Gamma-Secretase Complex mutations; however, the two diseases are not epidemiologically associated. Understanding the molecular differences between the two diseases may aid in the development of hypotheses for differing pathogenesis and ultimately, targets for detection.

**Aims:** To characterize the *in silico* structural and functional alterations to the Gamma Secretase Complex in documented mutations in Familial Hidradenitis Suppurativa, along with comparison of downstream substrate recognition and cleavage.

**Methods:** *In silico* analysis of publicly available genomic data, assessment of protein structure and binding affinity using Swiss-model and Dynamut was undertaken. Differential Expression was expressed using Log Fold Change using the general framework for linear models in R. Differentially expressed genes (DEGs) were defined by FCH ≥1.5 or ≤−1.5 and false discovery rate (FDR ≤ 0.05).

**Results:** Twenty three of 39 mutations in HS are degraded via nonsense mediated decay with altered substrate and binding affinity of substrates identified in the remaining mutations. Significant differential expression of ErbB4, SCNB1, and Tie1 in lesional skin was specific to Hidradenitis Suppurativa and EphB2, EPHB4, KCNE1, LRP6, MUSK, SDC3, Sortilin1 in blood specific to Familial Alzheimer's Disease.

**Discussion and Conclusions:** We present the first *in silico* evidence as to the impact of documented mutations in Familial Hidradenitis Suppurativa. We also demonstrate unique substrate recognition and cleavage between Hidradenitis Suppurativa and Familial Alzheimer's Disease, providing a potential explanation as to why the two diseases do not occur within the same pedigree. These proteomic signatures may be a first step in identifying reliable biomarkers for Familial Hidradenitis Suppurativa.

## Introduction

Familial Hidradenitis Suppurativa (HS) and Familial Alzheimer's Disease (AlzD) are two inherited diseases associated with mutations in the Gamma Secretase Complex (GSC) (1, 2). The GSC is a transmembrane protease composed of four subunits: presenilin-1 (*PSEN1)*, Nicastrin (*NCSTN*), anterior-pharynx-defective 1 (*APH-1*), and presenilin-enhancer-2 (*PEN-2*). Familial HS and AlzD are not epidemiologically associated (3) and no known mutations overlap between the two diseases (4), however the reasons why these two diseases do not co-occur (given that they are both associated with mutations in the GSC) is unknown.

The GSC cleaves up to 69 individual substrates (5), the most well-known being amyloid precursor protein (APP) associated with AlzD (6) pathogenesis. Of interest, altered GSC substrate proteolysis is seen in non-neural tissues (including cutaneous fibroblasts) in AlzD (6) suggesting that peripheral tissues such as cutaneous fibroblasts can be analyzed for diagnostic and predictive biomarkers of disease (6). The structural and functional impacts of GSC mutations in AlzD has been well-characterized through molecular dynamics *in silico* techniques, however, there is a lack of similar studies examining the role of GSC mutations in HS (7, 8).

Limited data exists assessing the impact of mutations on GSC proteolysis in HS epidermal keratinocytes (9), with existing data dependent upon the assumption that Notch signaling (the most studied GSC substrate in HS) is the sole pathogenic mechanism in the disease, which remains unproven (10). There is no known assessment of the impact of HS-associated mutations on other GSC substrates other than Notch (9, 10). Given the documented positive transcriptional feedback mechanisms in proteolyzed GSC substrates such as Notch (11), examining the differential expression of GSC substrates may give an indication as to the specific mechanistic pathways involved in each disorder. The lack of data regarding the effects of HS-associated GSC mutations impairs our ability to understand the molecular pathogenesis of HS, as well as accurately interpret novel peripheral biomarkers specific to HS vs. AlzD. It is also unclear what the normal background variation of GSC substrate expression is in the setting of cutaneous inflammation. This is important in order to interpret the functional significance of differential expression.

## Aims

We aimed to systematically assess all known mutations in the components of the GSC in Familial HS *in silico* for resulting protein structure and binding affinity. We also aimed to compare the downstream GSC substrate recognition and cleavage between HS and AlzD, along with a panel of other most common inflammatory dermatoses (psoriasis, atopic dermatitis, and alopecia areata) and neurodegenerative disorders (Parkinson's disease, Huntington's Disease) for comparison and identification of non-specific background effects.

## Methods

### Identification of Sequence Variants in Hidradenitis Suppurativa

Variants identified as pathogenic in our previous systematic review (4) were visually confirmed in the Integrative Genomics Viewer (IGV) version 2.4 (Broad Institute, Cambridge, Massachusetts, USA.). These reviews also assessed the pathogenicity of individual variants using pre-defined consensus criteria of the American College of Medical Genetics and Genomics and Association for Molecular Pathology (11).

### FASTA Amino Acid Sequences for Wild Type and Variants

FASTA amino acid sequences for wild type proteins in the GSC were sourced from UniprotKB/Swiss-Prot (www.uniprot.org) with the following Entries: Nicastrin (NCSTN): Q92542; Pre-Senilin 1 (PSEN1): P49768; Pre-Senilin 2(PSENEN): Q9NZ42.

### *In silico* Assessment of Protein Structure and Binding Affinity

Swiss-Model (www.swissmodel.expasy.org) was used in automated mode using FASTA format amino acid sequences to analyze protein conformational change. Those proteins without significant conformational alteration (based on visual inspection) were considered less likely to undergo nonsense mediated decay (NMD) and were then assessed for binding affinity. Dynamut (http://biosig.unimelb.edu.au/dynamut/) was employed using single mutation analysis. Protein Data Bank (PDB) structure of the combined gamma secretase complex was sourced from RCSB PDB (www.rscb.org) with PDB ID.

### Identification of Gamma Secretase Complex Substrates

A comprehensive list of GSC substrates was compiled from the existing literature with a total of 69 substrates identified (1).

### Gene Expression Data Sources-Skin

Publicly available gene expression data for skin were sourced from NCBI Gene Expression Omnibus (https://www.ncbi.nlm.nih.gov/) with the following GSE numbers:

- Hidradenitis Suppurativa Lesional Skin- GSE 72702 (*n* = 30).
- Psoriasis Lesional Skin- Krueger GSE 13355 (*n* = 122).
- Alopecia Areata Lesional Skin: GSE 45512 (*n* = 10).
- Atopic Dermatitis Lesional Skin GSE 32924 (*n* = 22).

Normal Unaffected Controls were pooled from GSE 13355, 45512, 32924 to use as a common reference. Non lesional samples (including GSE 72702) were excluded from analysis. General Hidradenitis Suppurativa gene expression data was used as no specific gene expression data is available for Familial HS patients.

### Gene Expression Data Sources-Blood

Publicly available gene expression data for whole blood were sourced from NCBI Gene Expression Omnibus (https://www.ncbi.nlm.nih.gov/) with the following GSE numbers and references:

- Hidradenitis Suppurativa Whole Blood: GSE 79149 (*n* = 26).
- Alzheimer's Disease Whole Blood: Mukhamedyarov et al. (12) (*n* = 10).
- Parkinson's Disease Whole Blood GSE 54536 (*n* = 10).
- Huntington's Disease Whole Blood: GSE 24250 (*n* = 14).

Normal Unaffected Controls were pooled from GSE 79149, GSE 54536, and GSE 24250 and reference 2 above to use as a common normal reference.

### Statistical Analyses

Statistical Analysis was performed using the standard R package (R Core Team, 2019). Differential gene expression between normal unaffected controls and HS, inflammatory dermatoses and neurodegenerative disorders was performed. Visualization of data was performed with GraphPad Prism 7 software (GraphPad Software). Expression values were modeled using a mixed-effects model with lesional categories as fixed factors and random effects for each patient. Fold Changes (FC) were estimated under the general framework for linear models in the R limma package with heteroscedasticity accounted for using parameter ArrayWeights. Batch effect was assessed and removed using the R limma package removeBatchEffect function. *P*-values from *t*-tests were adjusted for multiple hypotheses using Benjamini–Hochberg procedure. Differentially expressed genes (DEGs) were defined by FC ≥ 1.5 or ≤-1.5 and false discovery rate (FDR ≤ 0.05). Statistical comparison of substrate expression between conditions was conducted using one way ANOVA with *p* < 0.05 considered significant.

Further pathway analysis and assessment of upstream regulators was performed using the Ingenuity Pathway Analysis (IPA) Tool (Ingenuity H Systems, Redwood City, CA). Differentially expressed GSC substrates in HS and AlzD were analyzed to identify activated or suppressed biological pathways using IPA algorithms. Predicted activation scores (z scores >2 or <2) were considered significant and the description of pathways are based upon IPA algorithms and output.

## Results

**Figure 1:**
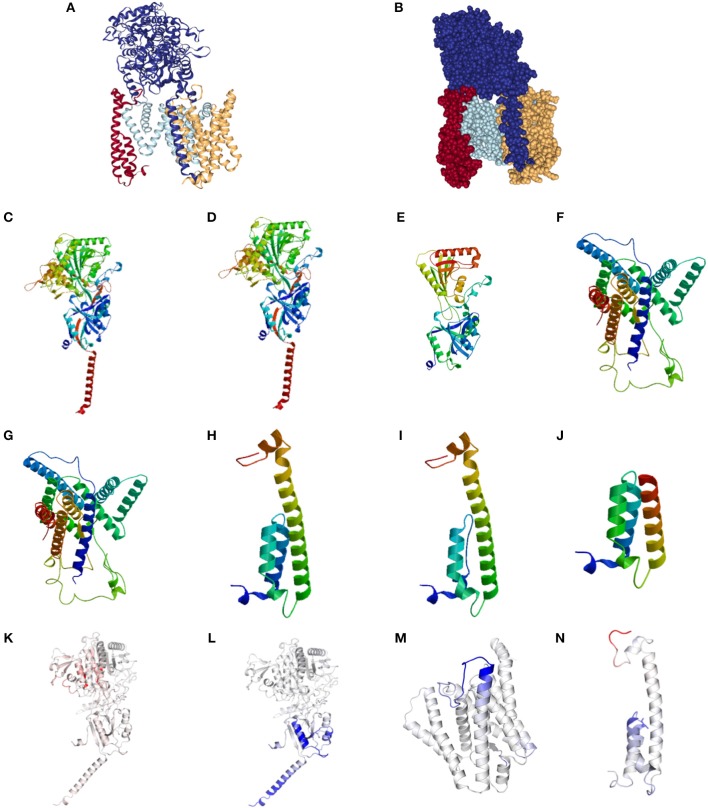
Structural and binding stability assessments of identified mutations in Familial HS. **(A)** Presents the structure of the gamma secretase complex (GSC) with Nicastrin (Dark Blue), PSEN1 (Light Blue), PSENEN (Red), and APH1 (Yellow). The “V” shaped transmembrane domain cleave site can be identified in light blue. Filled structure **(B)** demonstrates the binding pocket with access to the PSEN1 substrate cleavage site, surrounded by PSNEN and *NCSTN* substrate binding sites. Wild Type (WT) *NCSTN* **(C)**, *NCSTN* V75I **(D)**, and *NCSTN* Q420X **(E)**, PSEN1 WT **(F)**, *PSEN1* 953A>G **(G)**, *PSENEN* WT **(H)**, *PSENEN* 43_56del14 **(I)**, *PSENEN* 66delG **(J)**. Binding and affinity assessment of *NCSTN* 996+7G>A **(K)**, *NCSTN* V75I **(L)**, *PSEN1* 725delC **(M)**, *PSENEN* 66_67insG **(N)**. Blue indicates decreased binding affinity and increased flexibility with red indicating increased binding affinity with decreased flexibility. For comprehensive conformational alterations in AlzD the reader is referred to Berezovska et al. (13).

The normal structure of the GSC is presented in Figures 1A,B. Representative alterations found in HS in Nicastrin (Figures 1C–E), Pre-senilin 1 (Figures 1F,G), and Pre-senilin 2 (Figures 1H–J) demonstrating particular mutations with either minimal or significant structural alterations are shown. The complete list of structural alterations are presented in Supplementary Figures 1, 2. Structural analysis suggested 21 of 30 *NCSTN*, 0 of 3 *PSEN1* and 2 of 6 *PEN-2* mutations in HS are degraded via NMD (Supplementary Figures 1, 2).

Representative results of binding and substrate affinity alterations in HS in the 16 variants not degraded by NMD are presented in Figures 1K–N. The complete list is available in Supplementary Figure 3. Binding and flexibility alterations to the trans-membrane domain (TMD) of Nicastrin, Pre-senilin1, and Pre-senilin-2 were identified (Figures 1K–N), as well as binding alterations to potential substrate binding sites in the extracellular domains of the respective proteins (E3 for Pre-senilin 1 NTF, H6 for Nicastrin, A30 for Pre-senilin 2) (7, 14).

**Figure 2:**
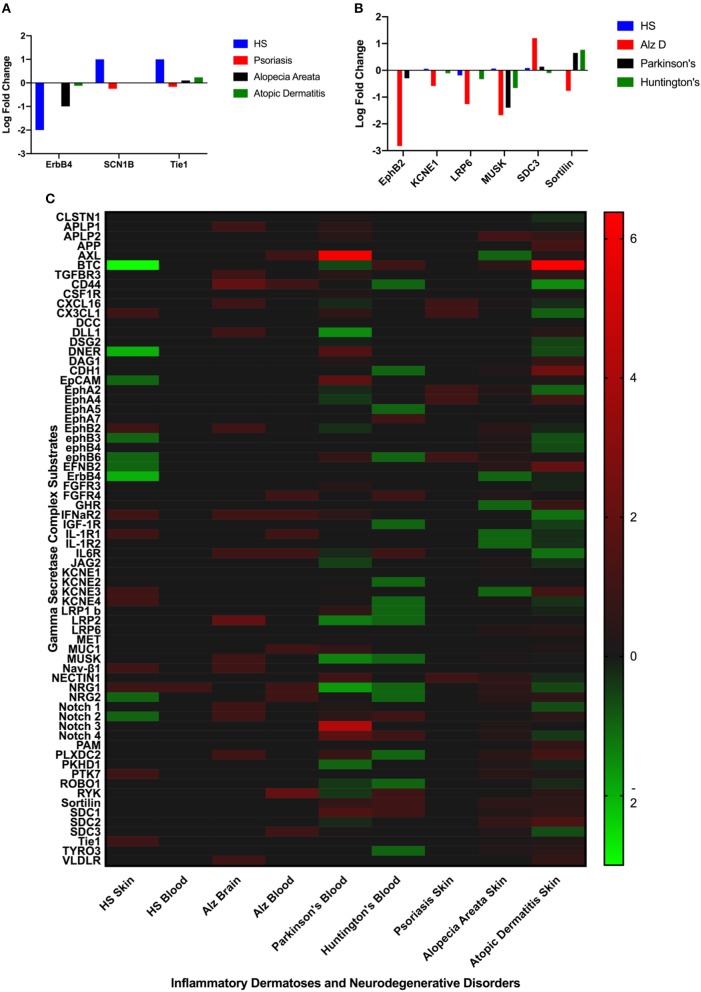
Comparison of significantly differentially expressed GSC substrates across inflammatory skin disease **(A)**, and neurodegenerative disorders **(B)**. Only the substrates specific to HS **(A)** or AlzD **(B)** are illustrated. Significant differential expression of ErbB4, SCNB1, and Tie1 in lesional skin was specific to Hidradenitis Suppurativa and EphB2, EPHB4, KCNE1, LRP6, MUSK, SDC3, Sortilin1 in blood specific to Familial Alzheimer's Disease. A Heatmap **(C)** of all GSC substrates across all measured datasets highlighting the non-specific differential expression in a number of disorders not associated with inherited mutations in the GSC.

Differentially expressed GSC substrates specific to HS and AlzD are presented in Figures 2A,B, and the complete heatmap of GSC substrate differential expression is presented in Figure 2C. Significant differential expression of ErbB4, SCNB1, and Tie1 in lesional skin was specific to HS and EphB2, EPHB4, KCNE1, LRP6, MUSK, SDC3, Sortilin1 in blood specific to AlzD. Other inflammatory dermatoses (psoriasis, alopecia areata, and atopic dermatitis) as well as neurodegenerative disorders (Parkinson's disease, Huntington's disease) included for comparison of non-specific background effects. Significant differential expression between HS and inflammatory dermatoses (*P* < 0.001), as well as AlzD and other neurodegenerative disorders (*p* < 0.05) was significant by one-way ANOVA.

In order to account for the possibility of differential cleavage of substrates altering the function (but not the total amount) of GSC substrates, we compared activated and suppressed pathways downstream of GSC substrate cleavage between AlzD and HS. Activated pathways associated with HS included apoptosis, “apoptosis of fibroblast cell lines,” “cell death of connective tissues,” “cell proliferation of fibroblasts,” and “binding of immune cells” as per Ingenuity Pathway Analysis. These were associated with the ErBB4, IFNAR1 IFNAR2, IL1R1 IL1R2, IGF1R substrates (The complete list of associated substrates associated with these pathways are available in Supplementary File 2). These pathways have previously been implicated in NCSTN knockdown cell lines, independently validating the results of our *in silico* methods (15). Activated pathways in AlzD included invasion of carcinoma cells, proliferation of connective tissues, invasion of tumor cells, and movement disorders (Supplementary File 2). These were associated with APP, CD44, AXL, CSF1R, MET, TGFBR3 substrates (Supplementary File 2). Common pathways which were differentially activated or suppressed in blood between AlzD and HS as inidicated in Ingenuity Pathway Analysis included: “advanced malignant tumor,” “cellular infiltration by leucocytes,” “metastasis of cell lines,” “movement disorders,” and “secondary tumors” (Supplementary Figure 4). (The complete list of substrates associated with these pathways are available in Supplementary File 2).

## Discussion

*In silico* analysis of HS-associated mutations in GSC identifies significant structural and functional alterations consistent with known sites of substrate binding and cleavage. Even in the setting of NMD of one component of the GSC, membrane localization of GSC is known to occur, albeit with altered proteolytic activity (14). NMD of NCSTN (as one of the most common results of HS associated mutations) is anticipated to increased substrate cleavage through the removal of the NCSTN extracellular domain “gatekeeper” (14) but may also reduce cleavage through the removal of extracellular substrate binding sites.

Unique HS-associated differential expression of GSC substrates was identified for ErbB4, SCN1B, and Tie1, although significant differential expression of multiple other substrates were seen in other inflammatory dermatoses. Given the lack of (known) altered GSC complex activity in these inflammatory disorders, this may indicate possible non-specific effects of inflammation upon expression and function of GSC substrates which warrants further investigation. This also brings into question whether alterations in substrates such as Notch are specific to disease (10), or rather a non-specific inflammation related finding. Similar non-specific background effects were also seen in neurodegenerative disorders such as Huntington's and Parkinson's Disease.

The identification of HS-specific substrates also raises the possibility that some of these substrates which are druggable targets (such as IGF-1R, Tie1) may represent novel therapeutic approaches. CSF1R, IFNAR1 IFNAR2, IL1R1 IL1R2 pathway upregulation is greater in HS than AlzD, consistent with its role in systemic inflammation. Interferon responsive pathways have been independently documented in NCSTN shRNA knockdown keratinocyte cell lines (16) supporting the role of these pathways in HS. Diabetes and obesity are also associated with HS (17), implicating IGF1R. Follicular hyperkeratinization, prominent dermal fibrosis and dermal tunnel formation in HS have led to hypotheses of Wnt signaling deficiencies in dermal fibroblasts and mesenchymal cells of the dermal papillae (18). FGFR4, MUC1, and MUSK pathway downregulation provide evidence to support this hypothesis. These results suggest that despite the inherent limitations of an *in silico* analysis of existing genomic data, this approach is capable of identifying key targets in disease.

The clinical validity of this work is supported by the fact that all assessed sequence variants are those deemed pathogenic through established criteria as published in our previous review (11), although a limitation to our study is that no external validation of functional confirmation has been undertaken to confirm these *in silico* findings. We note with interest that Notch was not identified as a HS-specific GSC substrate, despite the evidence from the published literature (19, 20) regarding alterations in Notch signaling and POGLUT1 (20), an endoplasmic reticulum O-glucosyltransferase involved in Notch signaling. This may indicate that Notch is not a HS- specific substrate and alterations in Notch and subsequent Notch-associated loci (21) may also be shared with AlzD. Further investigation into the role of Notch signaling across inflammatory dermatoses and neurodegenerative disorders may be informative in this regard.

## Conclusion

The results of our *in silico* analysis identify that HS-associated mutations have structural and potentially functional impact upon GSC substrates. These effects are distinct between HS and AlzD which explains their lack of co-occurrence in pedigrees. Our data identifies the differential expression of specific substrates, which may function as proteomic signatures of disease. Further prospective studies are needed to validate these targets. The downstream affected pathways confirm the previous experimental results of NCSTN knockdown cell lines (15) giving validation to our approach. Our results present a first step in understanding the molecular pathogenesis of Familial HS with a view toward diagnostic biomarkers of disease.

## Data Availability Statement

Publicly available datasets were analyzed in this study. This data can be found here (Supplementary File 1): Publicly available gene expression data for skin were sourced from NCBI Gene Expression Omnibus (https://www.ncbi.nlm.nih.gov/) with the following GSE numbers:

- Hidradenitis Suppurativa Lesional Skin- GSE 72702.
- Psoriasis Lesional Skin GSE 13355.
- Alopecia Areata Lesional Skin: GSE 45512.
- Atopic Dermatitis Lesional Skin GSE 32924.
- Hidradenitis Suppurativa Whole Blood: GSE 79149.
- Alzheimer's Disease Whole Blood: Mukhamedyarov et al. (12).
- Parkinson's Disease Whole Blood GSE 54536.
- Huntington's Disease Whole Blood: GSE 24250.

## Author Contributions

JF and KN designed the study. JF performed the analysis and wrote the manuscript. KN made revisions to the manuscript. All authors approved the final version of the manuscript.

### Conflict of Interest

The authors declare that the research was conducted in the absence of any commercial or financial relationships that could be construed as a potential conflict of interest.

## Supplementary Material

Supplementary Figure 1 Nicastrin morphological alterations by SWISS MODEL.

Supplementary Figure 2 PSEN1 and PSENEN morphological alterations by SWISS MODEL.

Supplementary Figure 3 Binding and flexibility assessment by dynamut for all proteins not undergoing NMD.

Supplementary Figure 4 Comparison of functional annotation for HS and Alzheimer's disease.

Supplementary File 1 Raw data of FCH and LogFCH in Gamma Secretase Substrates across HS, AlzD, Psoriasis, AD, Alopecia Areata, Parkinson's disease, and Huntington's disease.

Supplementary File 2 ADAM10 and ADAM17 expression between HS, AzD, Psoriasis, Atopic Dermatitis, Alopecia Areata, Parkinson's disease, and Huntington's disease.
