# Supplementary material for: In silico Analysis of Gamma-Secretase-Complex Mutations in Hidradenitis Suppurativa Demonstrates Disease-Specific Substrate Recognition and Cleavage Alterations
Source: Front Med (Lausanne). 2019 Sep 19;6:206. doi: 10.3389/fmed.2019.00206 (PMC6761225; doi:10.3389/fmed.2019.00206)
Supplement: Supplementary Figure 1 — Nicastrin morphological alterations by SWISS MODEL. [file Data_Sheet_1.PDF]

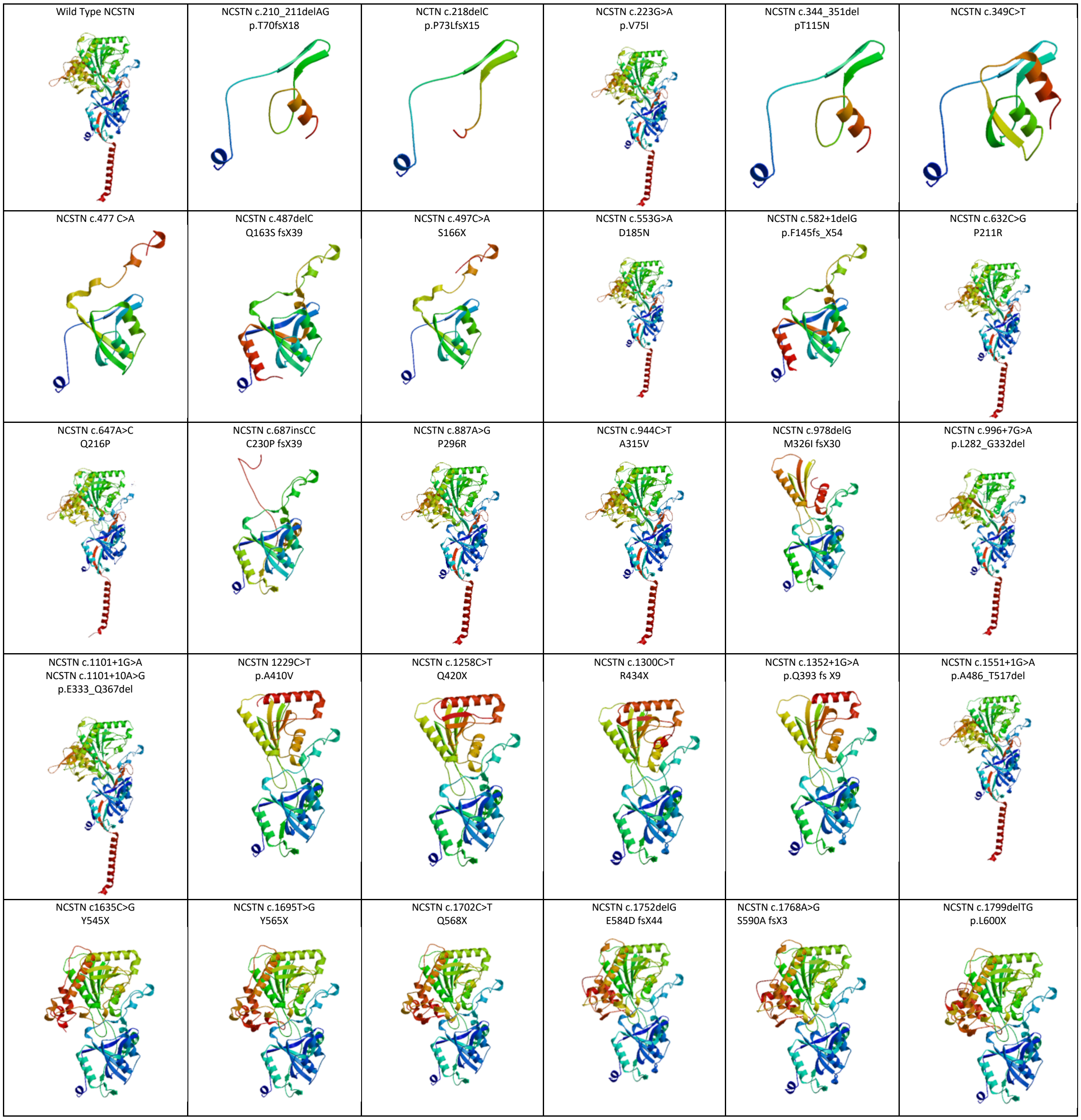
Wild Type NCSTN
NCSTN c.210_211delAG p.T70fsX18
NCTN c.218delC p.P73LfsX15
NCSTN c.223G>A p.V75I
NCSTN c.344_351del pT115N
NCSTN c.349C>T
NCSTN c.477 C>A
NCSTN c.487delC Q163S fsX39
NCSTN c.497C>A S166X
NCSTN c.553G>A D185N
NCSTN c.582+1delG p.F145fs_X54
NCSTN c.632C>G P211R
NCSTN c.647A>C Q216P
NCSTN c.687insCC C230P fsX39
NCSTN c.887A>G P296R
NCSTN c.944C>T A315V
NCSTN c.978delG M326I fsX30
NCSTN c.996+7G>A p.L282_G332del
NCSTN c.1101+1G>A NCSTN c.1101+10A>G p.E333_Q367del
NCSTN 1229C>T p.A410V
NCSTN c.1258C>T Q420X
NCSTN c.1300C>T R434X
NCSTN c.1352+1G>A p.Q393 fs X9
NCSTN c.1551+1G>A p.A486_T517del
NCSTN c1635C>G Y545X
NCSTN c.1695T>G Y565X
NCSTN c.1702C>T Q568X
NCSTN c.1752delG E584D fsX44
NCSTN c.1768A>G S590A fsX3
NCSTN c.1799delTG p.L600X
