# Supplementary material for: In silico Analysis of Gamma-Secretase-Complex Mutations in Hidradenitis Suppurativa Demonstrates Disease-Specific Substrate Recognition and Cleavage Alterations
Source: Front Med (Lausanne). 2019 Sep 19;6:206. doi: 10.3389/fmed.2019.00206 (PMC6761225; doi:10.3389/fmed.2019.00206)
Supplement: Supplementary Figure 2 — PSEN1 and PSENEN morphological alterations by SWISS MODEL. [file Data_Sheet_2.PDF]

PSEN1 and PSENEN Swiss Model

PSEN1 WT<br>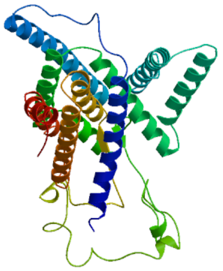	PSEN1<br>c.725delC<br>p.P242LfsX11<br>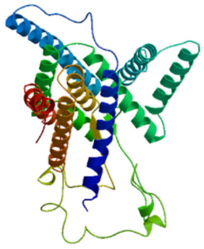	PSEN1<br>c.837+16G>T<br>p.N279G<br>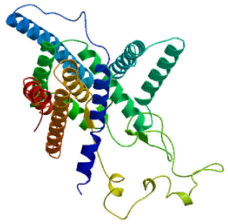	PSEN1<br>c.953A>G<br>p.E318G<br>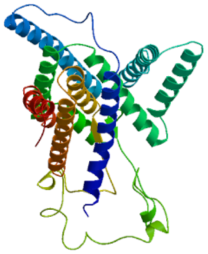
PSENEN WT<br>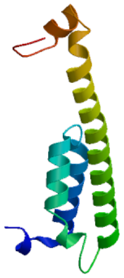	PSENEN<br>c.43_56del14<br>p.L15_F18del<br>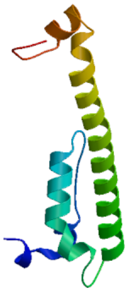	PSENEN<br>c.62-1G>C<br>p.F20_S55del<br>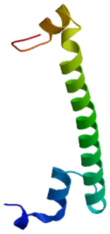	PSENEN<br>c.66delG<br>p.F23L fsX46<br>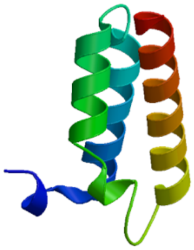
PSENEN<br>c.66_67insG<br>p.F23VfsX98<br>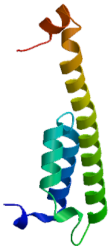	PSENEN<br>c.168T>G<br>p.Y56X<br>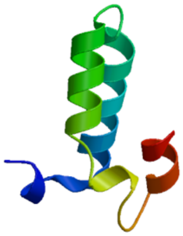	PSENEN<br>c.279delC<br>p. P94S fsX51<br>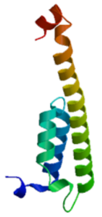	
