# Supplementary material for: In silico Analysis of Gamma-Secretase-Complex Mutations in Hidradenitis Suppurativa Demonstrates Disease-Specific Substrate Recognition and Cleavage Alterations
Source: Front Med (Lausanne). 2019 Sep 19;6:206. doi: 10.3389/fmed.2019.00206 (PMC6761225; doi:10.3389/fmed.2019.00206)
Supplement: Supplementary Figure 3 — Binding and flexibility assessment by dynamut for all proteins not undergoing NMD. [file Data_Sheet_3.PDF]

Binding Flexibility of Non-Degraded Protein Structures

NCSTN c.223G>A<br>p.V75I<br>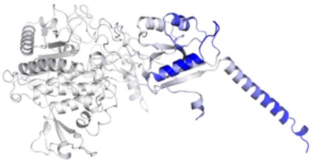	NCSTN c.553G>A<br>D185N<br>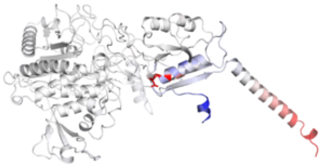	NCSTN c.632C>G<br>P211R<br>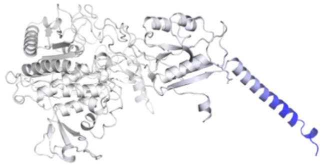
NCSTN c.647A>C<br>Q216P<br>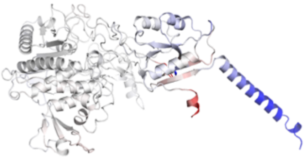	NCSTN c.887A>G<br>P296R<br>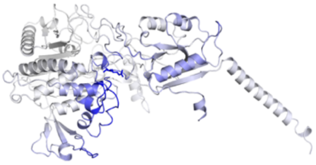	NCSTN c.944C>T<br>A315V<br>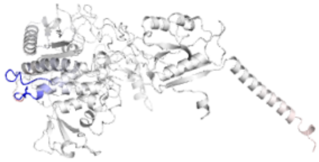
NCSTN c.996+7G>A<br>p.L282_G332del<br>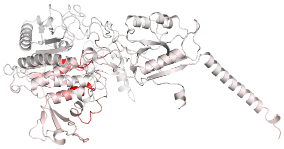	NCSTN c.1101+1G>A<br>NCSTN c.1101+10A>G<br>p.E333_Q367del<br>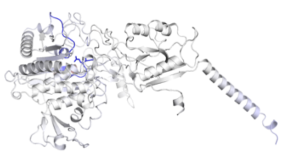	NCSTN c.1551+1G>A<br>p.A486_T517del<br>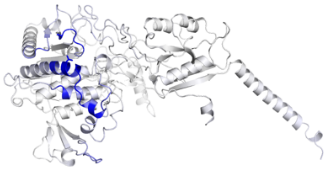
PSEN1<br>c.725delC<br>p.P242LfsX11<br>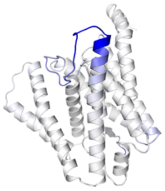	PSEN1<br>c.837+16G>T<br>p.N279G<br>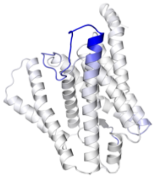	PSEN1<br>c.953A>G<br>p.E318G<br>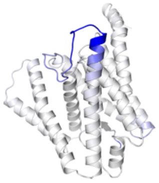
PSENEN c.43_56del14<br>p.L15_F18del<br>C Y<br>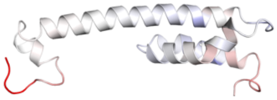	PSENEN<br>c.62-1G>C p.F20_S55del<br>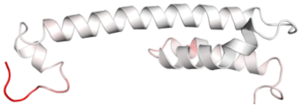	PSENEN<br>c.66_67insG<br>p.F23VfsX98<br>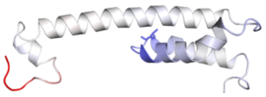
PSENEN<br>c.279delC<br>p. F94S fsX51<br>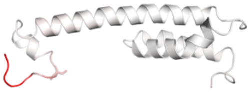		
