# Supplementary figures and images for: In silico Analysis of Gamma-Secretase-Complex Mutations in Hidradenitis Suppurativa Demonstrates Disease-Specific Substrate Recognition and Cleavage Alterations
Source: Front Med (Lausanne). 2019 Sep 19;6:206. doi: 10.3389/fmed.2019.00206 (PMC6761225; doi:10.3389/fmed.2019.00206)

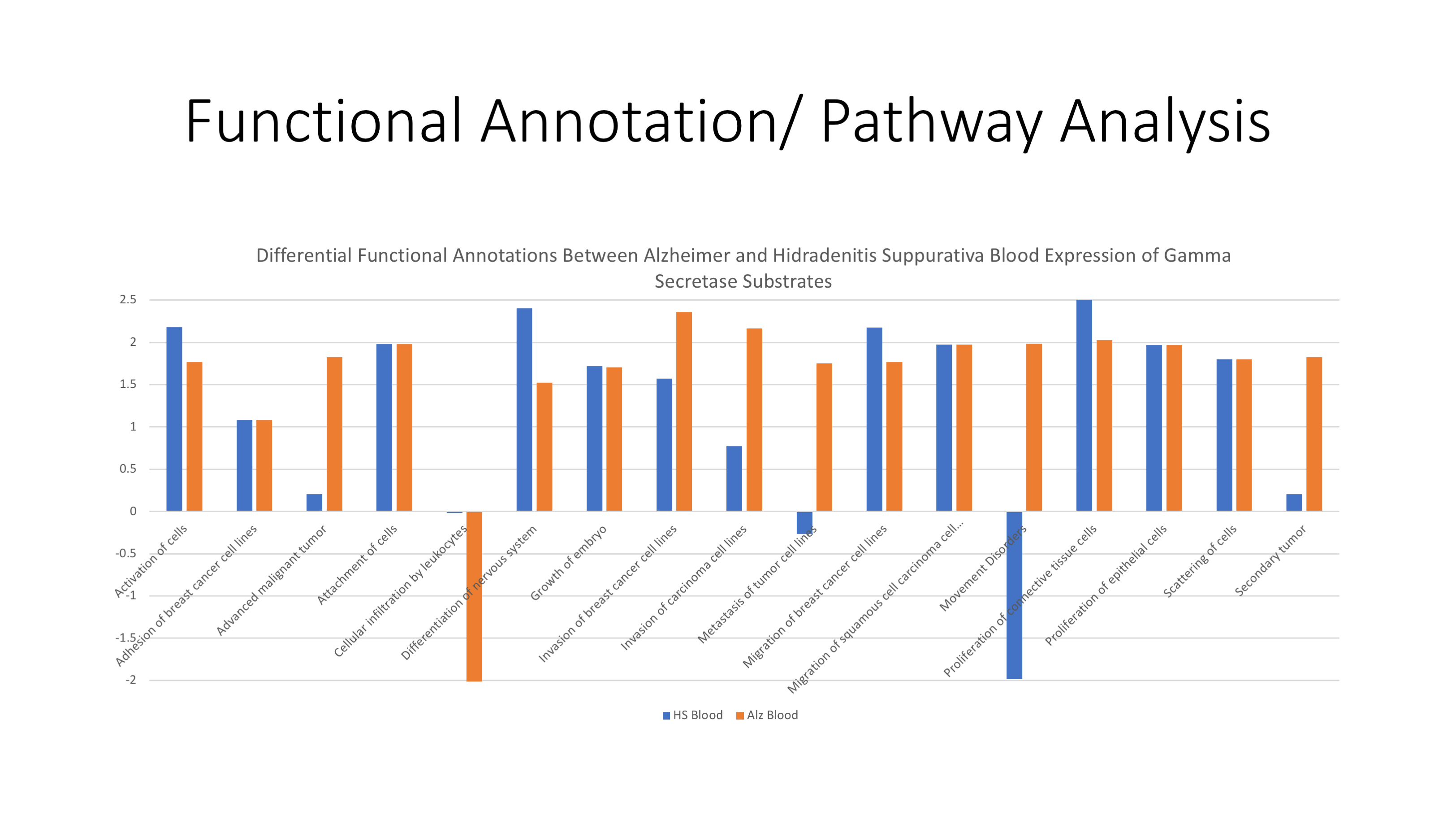

Supplement: Supplementary Figure 4 — Comparison of functional annotation for HS and Alzheimer's disease. [file Image_1.TIFF]
